# Influence of Rigor Mortis on Tendon Mobility in an Animal Fresh Cadaver Model

**DOI:** 10.3390/biology11101381

**Published:** 2022-09-22

**Authors:** Christoph Luecke, Marc Schnetzke, Christel Weiss, Stefan Studier-Fischer, Thorsten Guehring, Paul A. Gruetzner, Felix Porschke

**Affiliations:** 1 BG Trauma Center Ludwigshafen, Heidelberg University Hospital, Ludwig-Guttmann-Straße 13, 67071 Ludwigshafen, Germany; 2 German Joint Center, Atos Clinic, Bismarckstraße 9-15, 69115 Heidelberg, Germany; 3 Department of Medical Statistics, Medical Faculty Mannheim, University of Heidelberg, Theodor-Kutzer-Ufer 1-3, 68167 Mannheim, Germany; 4 Orthopedic Clinic Paulinenhilfe Diakonie-Klinikum Stuttgart, Rosenbergstraße 38, 70176 Stuttgart, Germany

**Keywords:** rotator cuff, rigor mortis, fresh cadaver study, biomechanic

## Abstract

**Simple Summary:**

In biomechanical research, fresh frozen cadaver material and embalmed specimens are often used to evaluate surgical approaches. Unlike, biomechanical research using fresh cadaver material is rare. There are no reliable data on the point of time when rigor mortis starts to have an impact on results with fresh cadaver material. In this study, the authors have conducted measurements using fresh porcine cadaver tendons of the supraspinatus muscle post mortem to determine the time of onset and the influence of rigor mortis on specimen tissue. 151 min post mortem, a significant decrease in tendon mobility was observed. Therefore, the authors of the presented study concluded that the onset of rigor mortis started 151 min post mortem and decreased the mobility of the tendon. Hence, biomechanical research using fresh cadaver material should ideally be conducted within 150 min post mortem to obtain in vivo-like results without being impacted by rigor mortis.

**Abstract:**

(1) Many biomechanical studies are performed using fresh frozen cadavers or embalmed specimens, although the biomechanical characteristics do not match the characteristics of in vivo tendons. Therefore, a fresh in vivo-like cadaver model has been introduced recently. As a limitation for studies with fresh cadavers, *rigor mortis* must be considered. The aim of this study was to evaluate the impact of the biomechanical properties and time of occurrence of *rigor mortis* in a fresh cadaver model. (2) For this study, 15 fresh porcine cadaver shoulders were used in an established biomechanical in vitro model to evaluate the onset of *rigor mortis*. Measurements took place at ten points of time (t1–t10) beginning 103 min post mortem (pm). The mobility of the supraspinatus tendon was measured in Newton (N) with a modified sensor-enhanced arthroscopic grasper. (3) The mean load measured at the time point t1 was 28.0 ± 11.2 N. The first significant decrease of mobility occurred 151 min post mortem (t4) at a mean load of 30.2 ± 13.7 N. From 227 min pm to 317 min pm, there was no further significant increase. (4) Tendon mobility decreases significantly within the first three hours after the killing. Therefore, reliable results can be obtained within 150 min post mortem before the onset of *rigor mortis* alters the biomechanical properties.

## 1. Introduction

The mobility of tendons concerning muscle–tendon complexes is an increasingly discussed and investigated topic. The mobility of the tendon seems to determine the success and the outcome after rotator cuff repair [1,2,3,4]. To achieve a better understanding of the characteristics of tendons, research on in vivo and in vitro models is necessary. Often, such research is performed using fresh frozen cadavers or embalmed specimens in an in vitro setting [5,6,7,8].

Compared with in vivo tendons and muscles, one disadvantage is an altered behavior of the muscle–tendon complex of freshly frozen cadavers or embalmed specimens [9,10,11,12]. Fresh frozen tendons are stiffer and lead to different results in biomechanical testing in comparison to fresh tendons [10,11,12]. Clavert et al. [10] compared the long head of human *biceps brachii* tendons in fresh and frozen states under load. They observed a difference in failure properties and a lower elasticity for the frozen state [10]. Using a canine model Gottsauner et al. [11] showed that the load–displacement curves between fresh and frozen specimens differed from each other. Frozen bone-muscles complexes showed a lower stiffness and load to failure [11]. Therefore, Porschke et al. [13] introduced a fresh cadaver model recently showing reliable measurements and in vivo-like biomechanical properties within a short period post mortem. It was demonstrated that the porcine *supraspinatus* tendon produces a typical load–displacement curve under lateralization with the same characteristic behavior shown in in vivo and in vitro animal models for the *supraspinatus* tendon [14,15]. Further, it was shown that the mean load under lateralization using the fresh cadaver model was similar to the intraoperative repair tension of in vivo human *supraspinatus* tendons [13,16,17].

The onset of *rigor mortis* for measurements over a longer period post mortem can be a possible bias of a fresh cadaver model. The literature lacks sufficient data on the timing when *rigor mortis* starts to alter the biomechanical properties of the muscle–tendon complex. Only a few studies investigate the biomechanical behavior and microscopic changes of the muscle–tendon complex influenced by *rigor mortis* [18,19,20,21,22]. The aim of this study was to evaluate the impact of *rigor mortis* on the biomechanical properties and to determine the time of occurrence of *rigor mortis* in a fresh cadaver model.

## 2. Materials and Methods

### 2.1. Specimen and Preparation

For this study, fifteen fresh porcine cadaver shoulders were evaluated using a validated biomechanical model [13]. The shoulders were harvested from 15 domestic pigs (*Sus scrofa forma domestica*) with an age of 6–8 months. In accordance with the national law on animal protection, no approval for the study was needed, since the animals were killed for the purpose of the food industry using a captive bolt gun. A healthy domestic porcine donor with an age of 6–8 months was an inclusion criterion. Obvious damage to the glenohumeral joint, preexisting defect of the rotator cuff and tendon slippage within the measurement were exclusion criteria.

After the killing, the shoulders were disarticulated from the thorax, avoiding any damage to the rotator cuff and deltoid muscle. The elbow joint was exarticulated and *biceps* and *coracobrachialis* muscles were resected distally at the height of the elbow joint. Skin and subcutaneous fat were removed completely. The lateral part of the deltoid was detached from the humerus over 2 cm allowing access to the supraspinatus insertion.

Mobility measurements were conducted using a custom-designed testing station which was described in detail in a recent study [13]. In brief, the *scapula* was fixated, by three screws on a mounting plate. Contact pressure between the *subscapularis* and the mounting plate was avoided by spacers. In addition, the *humerus* was set in place at 30° of abduction using another screw.

The humeral *supraspinatus* insertion was detached completely from the footprint to mimic a complete full-thickness non-retracted rotator cuff tear. Tendon proportions were measured using a digital caliper.

Throughout the whole preparation process and measurement period, the ambient temperature was kept constant at 20 °C ± 0.5, and humidity at 45% ± 2.5. For harvesting, preparation and cyclic preloading (10 min) in total 103 min elapsed between killing and the first measurement. 

### 2.2. Measurement of Mobility

Mobility of the supraspinatus tendon muscle complex was evaluated using a custom-designed testing station by measuring the force applied to lateralize the tendon.

**Figure 1:**
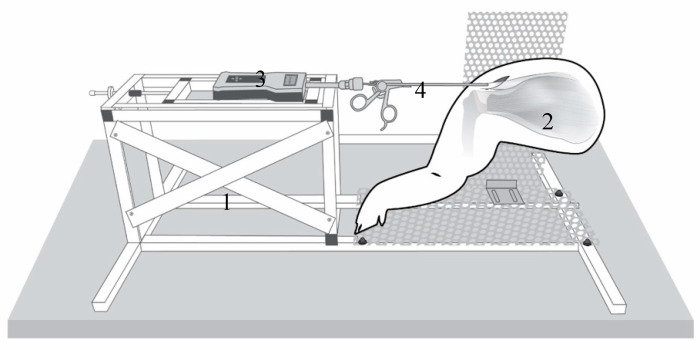
Testing station (1) with the porcine shoulder (2): The industrial force gauge (PCE-FB 200, accuracy ±0.1%, precision 0.05 N, measuring range 0.5–200 N, Fa. PCE Instruments) (3) connection to a commercial arthroscopic grasper (4) (Rotator cuff grasper: 4.2 mm shaft diameter with traumatic jaws, and a self-releasing locking mechanism from Fa. Arthrex Inc., Naples, FL, USA) modified by a custom-made aluminum fast adapter (Fa. Surgitaix, Herzogenrath, Germany).

The setup for testing is shown in Figure 1 (Figure 1). The tendon is preloaded with 0.1 N to define the starting point (0 mm).

The measurement of mobility was then performed by lateralizing the tendon in 1 mm increments. At each step (1 mm) and after an equilibrium period of 5 s, the load in Newtons was registered by the sensor-enhanced, arthroscopic grasper (SEAG). Each measurement process was completed at 12 mm lateralization. Tendon mobility was defined as the force applied at maximum lateralization (12 mm) (mean of three consecutive test cycles). The force measured and the mobility of the tendon are inversely related to each other.

### 2.3. Test Protocol

#### 2.3.1. Test Cycle

Before the measurements started, the myotendinous complex was preconditioned with ten cycles of 12 mm lateralization (1 mm/5 s). Every test cycle consisted of three measurement processes. After reaching the endpoint (12 mm lateralization), the SEAG was set back to the starting point (0 mm lateralization) and, after a 4-min resting period, the measurement was repeated in the same manner twice.

#### 2.3.2. *Rigor Mortis* Evaluation

**Figure 2:**
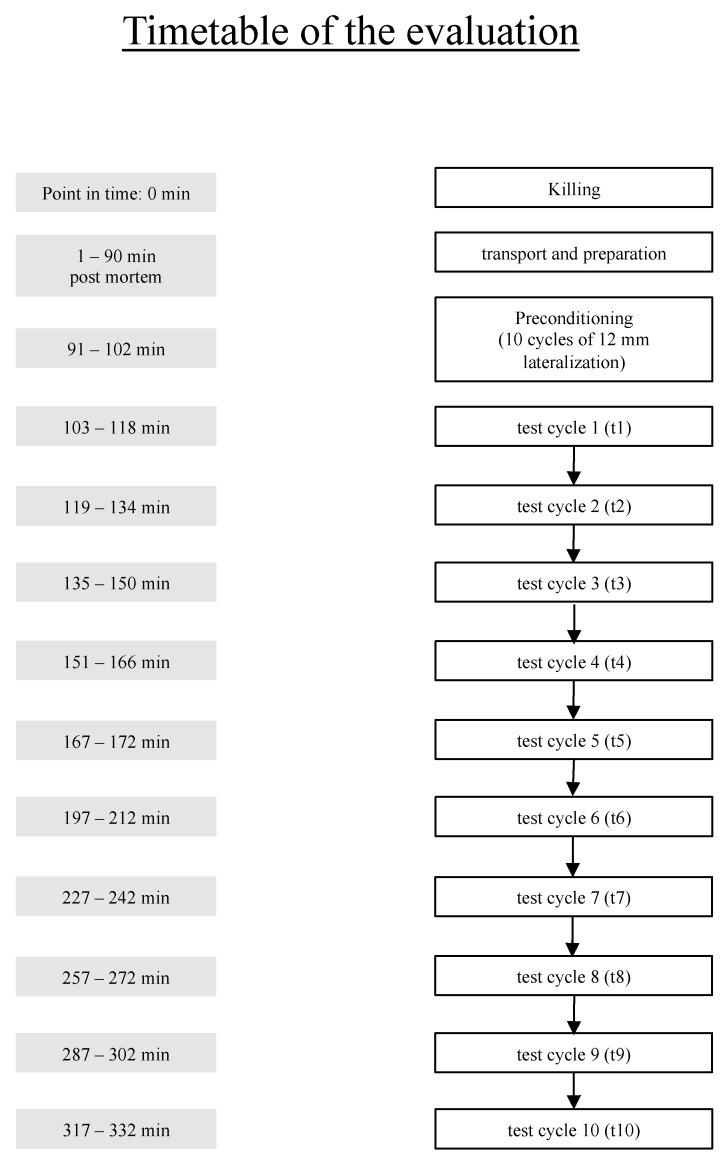
Time schedule post mortem.

For evaluation of *rigor mortis*, the tendon mobility was tested repeatedly beginning 103 min post mortem (t1) to 317 min post mortem (t10). Within the first 167 min mobility was tested every 15 min, and afterwards every 30 min (Figure 2).

### 2.4. Statistics

Statistical analysis was performed by a biometrician from the Medical Faculty Mannheim of Heidelberg University, using the statistical software SAS, Release 9.4, (SAS Institute, Cary, NC, USA).

For quantitative variables, mean and standard deviations were calculated.

Changes in mobility over time were evaluated using an ANOVA for repeated measurements with post hoc testing according to Tukey–Kramer using the SAS procedure PROC MIXED. In general, a test result was considered statistically significant if the corresponding *p*-value was less than 0.05.

## 3. Results

A total of 15 shoulders was included (7 left and 8 right shoulders). None of the samples had to be excluded from the study. The mean width of the tendon stump was 11.2 mm ± 2.3. The mean length of the myotendinous unit was 44.5 mm ± 3.5. The mean force for complete lateralization (tendon mobility) in native tendons was 32.1 N ± 14.6. Neither tendon stump width nor length of the myotendinous unit did affect the tendon mobility (*p* = 0.608; *p* = 0.972).

Tendon mobility significantly decreased throughout the testing period (t1: 28.0 ± 11.2 N vs. t10: 34.3 ± 13.3 N; *p* < 0.0001). In detail, the post hoc testing revealed no significant changes within the first 135 min of testing (t1 vs. t3). After 151 (t4) minutes post mortem, the mobility decreased significantly (t1 vs. t4, *p* = 0.030). Afterwards, mobility continuously decreased over time until 227 min post mortem (t7) (Table 1).

**Figure 3:**
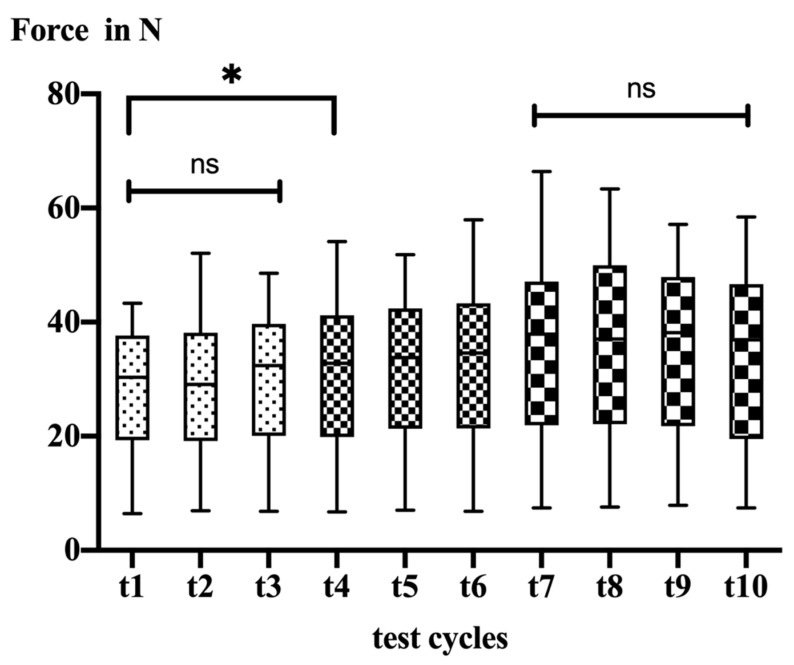
Box plots illustrating data of all test cycles for 12 mm lateralization; ns = not significant, * = significant difference.

After 227 min (test cycle 7), there was no further significant decrease in tendon mobility (Figure 3).

## 4. Discussion

The aim of this study was to evaluate the impact of *rigor mortis* on the biomechanical properties and to determine the time of occurrence of *rigor mortis* in a fresh cadaver model. The major finding of this study is that *rigor mortis* significantly alters the biomechanical properties of the muscle–tendon complex after 151 min post mortem.

From the beginning of testing until 135 min pm (t3), no significant change in the mobility of the muscle–tendon complex was observed. The first significant loss in mobility occurred 151 to 166 min pm (t4). After 227 min post mortem (t7) there was no further significant change in mobility of the muscle–tendon complex. Rather, the mean absolute measured mobility between 227 min and 317 min post mortem (t7 to t10) seemed to remain unchanged. The results of the current study suggest an onset of *rigor mortis* between 151 min and 227 min post mortem (t4 to t7), afterwards no further significant change of mobility occurred.

Using *tibialis anterior* muscle of New Zealand White rabbits, Van Ee et al. [21] found no significant difference in the immediate post mortem response to the passive live response during the first eight hours of biomechanical testing. The biomechanical results of Van Ee et al. [21] are different from the results of this study with regard to the time of occurrence of *rigor mortis*. The different results may be explained by the different schedules with only hourly elongation of the muscle–tendon complex and using smaller specimen. In contrast to Van Ee et al. [21], Kobayashi et al. [22] showed that the measured maximum tension of the muscle caused by *rigor mortis* for red muscle at 25 °C occurred 186 ± 12 min after death. These results are similar to the results of this study. In difference to Kobayashi et al. [22], in this study a whole porcine muscle–tendon complex was tested at 20 ± 0.5 °C ambient temperature. Kobayashi et al. [22] found out that the onset of *rigor mortis* is slower at lower temperatures of the muscle and in white muscle tissue than in red muscle tissue. These different results show that the onset of *rigor mortis* depends on many variables such as specimen size, tissue, temperature, and species. Therefore, a comparison of the results without controlling for the above-mentioned variables is not recommended.

Biomechanical in vitro models are important to validate and evaluate certain therapy concepts, e.g., surgical approaches. In order to produce reliable results and preferably similar results as in vivo testing, the used tissue should ideally behave like in vivo material. Gottsauner et al. [11] found evidence that fresh frozen muscle–tendon complexes show a lower load to failure in biomechanical testing than fresh tendons. Leitschuh et al. [12] showed that fresh frozen muscle–tendons complexes behave differently under load compared to fresh cadaver muscle–tendon complexes. Clavert et al. [10] presented similar results as Leitschuh et al. [12] by comparing fresh frozen human tendons to a fresh cadaver tendon. Overall, these results suggest that fresh frozen muscle–tendon complexes are inferior to fresh cadaver tissue for biomechanical testing [10,11,12]. This is in line with the results of this study for the test cycles after the onset of *rigor mortis*. At 227 min pm (t7), the mobility of the muscle–tendon complex is significantly lower to the previous test cycles though the elongation is the same.

This study confirmed the change in biomechanical behavior of the muscle–tendon complex during the post mortem period. The mobility of the muscle–tendon complex significantly decreased post mortem. Within the first 150 min after death, *rigor mortis* is not relevant in the biomechanical testing of fresh cadaver muscle–tendon complexes. This is an important finding as it shows that fresh cadaver muscle–tendon complexes could be used within 150 min post mortem. On the contrary, results obtained after 150 min post mortem from fresh cadaver models are most likely to be influenced by the occurrence of *rigor mortis*.

Nevertheless, there are limitations to the approach of this study. The biomechanical testing took place in an in vitro setting. The direct conversion of this testing into an in vivo model is limited due to the setup of testing. Further studies are needed to compare fresh cadaver models with in vivo models. Further, the results are collected from porcine cadaver shoulders. A direct translation into an in vivo and the human setting is not possible. Using the shoulder with *scapula,* rotator cuff, deltoid muscle, *humerus* and portions of the *biceps* and *coracobrachialis* instead of the full body may alternate the onset of *rigor mortis.* As shown by Kobayashi et al. [22], the onset of *rigor mortis* occurs at a slower rate when temperatures are low. A full body takes longer to cool down at ambient temperature. Therefore, the onset of *rigor mortis* in this model may be slower than in a full-body model. For reliable and comparable results, the use of body parts instead of a full body is recommended. Another limitation is the duration of the testing. Testing was completed within four hours. Another study revealed the onset of mortis not until eight hours post mortem, which may indicate that the testing period was too short to observe the onset of *rigor mortis* [21]. However, the presented study revealed a steady state of biomechanical properties after 227 min without any significant changes over 90 min. In fact, the aim of our study was to evaluate the impact of *rigor mortis* on the biomechanical properties and time of occurrence of *rigor mortis* in a fresh cadaver model. This was found after approximately two and a half hours post mortem. Therefore, we suggest that four hours of measurements are sufficient.

In this study, tendon mobility decreased significantly two and a half hours after death. Therefore, a timeframe of approximately 150 min post mortem exists for obtaining reliable results before the onset of *rigor mortis* alters the biomechanical properties.

## 5. Conclusions

The study findings indicate that there is a timeframe of approximately 150 min post mortem in a fresh cadaver in vitro model to obtain reliable in vivo-like results. The *rigor mortis* influences the biomechanical behavior of the muscle–tendon complex 150 min post mortem.

## Figures and Tables

**Table 1:** Measured force per test cycle.

Time pm (Test Cycle)	Force in N	Standard Deviation in N
103 min (t1)	28.02	11.19
119 min (t2)	28.99	13.00
135 min (t3)	29.63	12.96
151 min (t4)	30.24	13.70
167 min (t5)	31.08	13.87
197 min (t6)	32.48	14.78
227 min (t7)	35.54	16.30
257 min (t8)	35.78	16.49
287 min (t9)	35.08	15.62
317 min (t10)	34.28	16.26

## Data Availability

Some or all data and models that support the findings of this study are available from the corresponding author upon reasonable request.
